# Are Aptamer-Based Biosensors the Future of the Detection of the Human Gut Microbiome?—A Systematic Review and Meta-Analysis

**DOI:** 10.3390/bios14090423

**Published:** 2024-09-02

**Authors:** Maria João Moreira, Manuela Pintado, José M. M. M. De Almeida

**Affiliations:** 1 CBQF—Centro de Biotecnologia e Química Fina—Laboratório Associado, Escola Superior de Biotecnologia, Universidade Católica Portuguesa, Rua Diogo Botelho 1327, 4169-005 Porto, Portugal; mpmoreira@ucp.pt (M.J.M.); mpintado@ucp.pt (M.P.); 2 INESC TEC—Institute for Systems and Computer Engineering, Technology and Science, University of Porto, 4169-007 Porto, Portugal; 3 Department of Physics, School of Sciences and Technology, University of Trás-os-Montes e Alto Douro, 5001-801 Vila Real, Portugal

**Keywords:** aptamers, biosensors, gut microbiota, optical sensors, chemical sensors

## Abstract

The gut microbiome is shaped early in life by dietary and lifestyle factors. Specific compounds in the gut affect the growth of different bacterial species and the production of beneficial or harmful byproducts. Dysbiosis of the gut microbiome has been linked to various diseases resulting from the presence of harmful bacteria and their byproducts. Existing methods for detecting microbial species, such as microscopic observation and molecular biological techniques, are costly, labor-intensive, and require skilled personnel. Biosensors, which integrate a recognition element, transducer, amplifier, signal processor, and display unit, can convert biological events into electronic signals. This review provides a comprehensive and systematic survey of scientific publications from 2018 to June 2024, obtained from ScienceDirect, PubMed, and Scopus databases. The aim was to evaluate the current state-of-the-art and identify knowledge gaps in the application of aptamer biosensors for the determination of gut microbiota. A total of 13 eligible publications were categorized based on the type of study: those using microbial bioreceptors (category 1) and those using aptamer bioreceptors (category 2) for the determination of gut microbiota. Point-of-care biosensors are being developed to monitor changes in metabolites that may lead to disease. They are well-suited for use in the healthcare system and offer an excellent alternative to traditional methods. Aptamers are gaining attention due to their stability, specificity, scalability, reproducibility, low production cost, and low immunogenicity. While there is limited research on using aptamers to detect human gut microbiota, they show promise for providing accurate, robust, and cost-effective diagnostic methods for monitoring the gut microbiome.

## 1. Introduction

Trillions of microbial cells are present in the gut, proximally 10^14^–10^15^, contributing to immune function, nutrient processing, and other aspects. The gut microbiome (GM) is constituted by *Firmicutes*, *Bacteriodetes*, *Actinobacteria*, *Fusobacteria*, *Proteobacteria*, *Tenericutes*, *Verrucomicrobiota*, and *Euryarchaeota* phylum [1]. The *Firmicutes* (65%) and *Bacteroidetes* (23%) are the most abundant species, and their ratios are used to perceive GM disproportions, known as dysbiosis [2]. The presence of harmful microorganisms is linked to a wide range of diseases, specifically cancer, cardiovascular, and neurodegenerative diseases [3]. The gut microbiota produces harmful and beneficial metabolites that can contribute to the development of disease or provide protection [4,5]. Moreover, it helps in food decomposition, fermentation of complex carbohydrates, amino acids, and lipid-protein digestion [6]. Short-chain fatty acids (SCFAs) such as acetate, propionate, butyrate, and gamma-aminobutyric acid (GABA) play a crucial role in the prevention of several diseases and gut-brain communication [7,8]. Moreover, it is possible to regulate oxidative stress with other metabolites such as lipopolysaccharides, tryptophan, and polyphenolic metabolites [9]. On the other hand, p-cresol-sulfate, indoxyl sulfate, deoxycholic acid, lithocholic acid, tyramine, trimethylamine-N-oxide, trimethylaminuria, and N-phenylacetylglutamine metabolites in certain amounts can be associated with several diseases, for example, neurodegenerative and cardiovascular diseases [3,10]. Thus, it is necessary to develop fast methods applied in real time to estimate the influence of basal microbiota, fermentability, and specificity of current and potential prebiotics. DNA sequencing, high-performance liquid chromatography, and gas chromatography have been used to perceive GM composition [11]. Nevertheless, they have several disadvantages, especially because they require highly qualified employees to execute laboratory procedures on sophisticated and expensive machinery, subsequently not suitable in real-time [12,13]. A biosensor is an analytical device that integrates a recognition element, transducer, amplifier, signal processor, and display unit, permitting conversion of a biological event into an electronic signal [14]. They are being produced for diverse important applications, specifically in the healthcare sector, industrial research, food safety control, and other commercial sectors (agricultural, environmental governance) [15]. Nowadays, point-of-care biosensors are being developed to help control alterations of several metabolites in the human body, which can originate aging-related diseases [16,17]. This review aims to provide guidance on the design and use of aptamer biosensors for detecting microorganisms and their metabolites in the gastrointestinal tract. A systematic review of the scientific literature was conducted to assess the status of gut microbiota detection using different biosensors. The insights derived from this review shed light on the methodologies employed in these studies and pinpoint areas of knowledge gaps that require attention in the future.

## 2. Links between the Gut Microbiome, Their Metabolites, and Diseases

Food habits, physical activity, age, genetics, ethnicity, medications, environmental conditions, lifestyle, immune function, and well-being factors determine the fecal microbial community and their intervention in human health status [18]. Besides, the type of diet modifies the gut microbiota; for example, the presence of high fiber and animal protein increases *Bacteroidetes*, and the presence of higher fiber carbohydrates increases *Firmicutes* [5,6,19]. Dysbiosis of the GM has been associated with several diseases caused by harmful bacteria and their metabolites [3,9,20]. The Firmicutes is constituted by several genera, such as *Lactobacillus*, *Roseburia*, *Eubacterium*, *Faeculabecteriam*, *Lachnospiracea, Rumonicoccus*, *Acidaminoccocus*, *Romboutsia*, *Lactococcus lactis*, *Lactobacillus plantarum*, *Streptococcus bovis* and *Enterococcus faecalis*. *Bacteroides fragilis* and *Prevotella* belong to the Bacteroidetes phylum. The most abundant genus of Actinobacteria is *Bifidobacterium* [2,21]. *Helicobacter pylori* and *Escherichia coli* (*E. coli*) belong to the Proteobacteria phylum. *S. bovis*, *Enterococcus faecalis*, *H. pylori*, *E. coli*, *B. fragilis*, *Clostridium septicum*, *and Fusobacterium* spp. are associated with cancer, cardiovascular, and neurodegenerative diseases [3,22,23,24,25]. Beneficial gut microbiota such as *Akkermansia muciniphila*, *Bifidobacterium*, *Lactobacillus*, *Roseburia*, *Eubacterium*, and *Faecalibacterium* are the most important species for the treatment of diseases associated with dysbiosis [3,22,23,24,25,26,27,28]. During fecal fermentation, the gut microbiota can produce short-chain fatty acids together with gas (H_2_ and CO_2_). The concentrations of SCFAs are altered in certain diseases, specifically butyrate, propionate, and acetate [8]. Normally, they constitute approximately 90–95% of the total SCFAs in a molar ratio of 60:20:20, with concentration proximally from 50 to 150 mM in the human gut. Valeric acid and caproic acid are considered fundamentals for maintaining metabolic balance through immune modulation, anti-inflammatory properties, and improved carbohydrate and lipid metabolism [29]. Butyrate is crucial for colonic barrier function, whereas acetate is an energy substrate for the brain, liver, and muscles [30]. Moreover, the microbial community generates other metabolites, specifically neurotransmitters and their precursors, such as dopamine, trimethylamine, phenylacetic acid, lipopolysaccharide, flagellin, peptidoglycan, GABA, acetylcholine, bile acid, norepinephrine, monoamine, and branched-chain amino acids [4,10]. *Lachnospiraceae*, *Ruminococcaceae*, *Acidaminococcaceae* spp., *Roseburia*, and *Faecalibacterium* are butyrate producers, with the acetyl-coenzyme A pathway being the most prevalent. *Roseburia* and *Faecalibacterium* have shown anti-inflammatory and anti-obesity effects, as well as immune system modulation [30]. Besides, *Streptococcus*, *Prevotella*, *Bifidobacterium*, and *Clostridium* spp. are acetate producers, and propionate is synthesized by *Enterococci* and *Veillonella* species [2]. *Clostridium* species may generate butyrate from acetate and lactate [31]. *Bacillus* and *Escherichia* species synthesize dopamine in the gut microbiota [32]. Moreover, GABA is produced by *Levilactobacillus*, *Lactococcus*, and *Bifidobacterium*, and has been shown to improve sleep and resilience to mental stress [33]. The production of serotonin is facilitated by several species, explicitly, *E. coli*, *Lactococcus lactis* subsp. *cremoris*, *Lactobacillus plantarum*, *Candida* spp., *Streptococcus* spp., *Streptococcus thermophilus*, *Escherichia* spp., and *Enterococcus* spp. [10]. In summary, the gut microbiota and its metabolites play a significant role in human health and disease, and understanding their composition and functions is crucial for developing targeted interventions and treatments. Therefore, the GM modifies throughout life, producing several metabolites that can cause aging-related diseases, making it crucial to develop fast, accurate, and inexpensive point-of-care and clinical diagnostic technologies.

## 3. Materials and Methods

**Figure 1:**
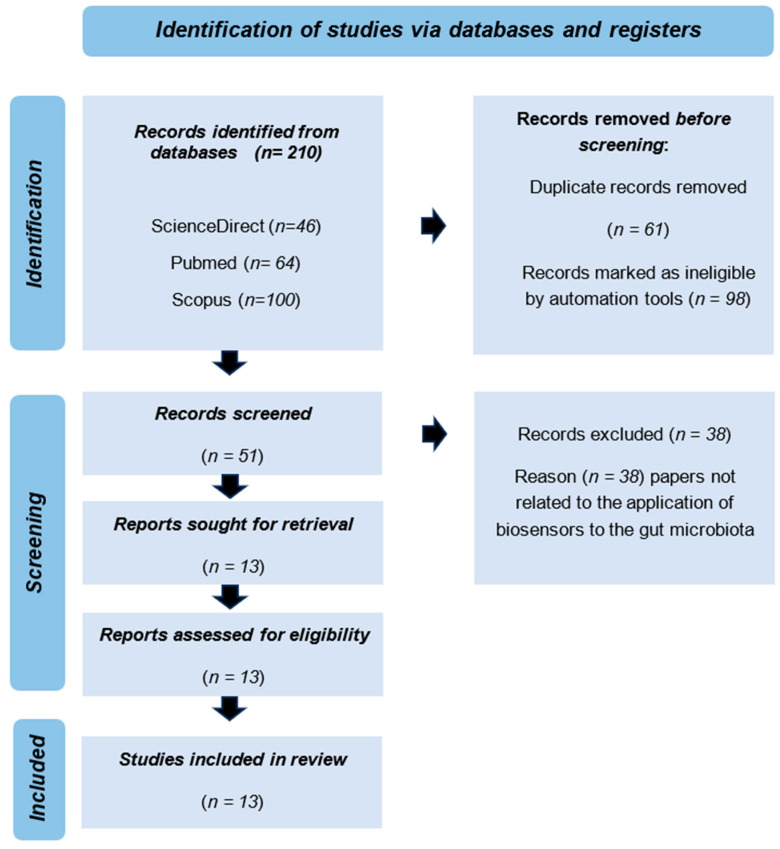
Schematic review selection process performed according to the PRISMA 2020 flow diagram.

A comprehensive search of scientific literature was conducted by two reviewers to find and evaluate publications related to biosensors applied to the detection of gut microbiota and their metabolites. The study followed the guidelines of the Preferred Reporting Items for Systematic Reviews and Meta-Analyses to ensure that the final report is transparent, consistent, and reliable [34]. Figure 1 presents a flow diagram summarizing the literature search process, screening, and selection of potential studies. 

Three databases, ScienceDirect (www.sciencedirect.com (accessed on 1 May 2024)), PubMed (https://pubmed.ncbi.nlm.nih.gov/ (accessed on 5 May 2024)), and Scopus (www.scopus.com (accessed on 10 May 2024)), were utilized to access scientific publications on the detection of gut microbiota with biosensors. A comprehensive search based on the query ((gut) AND (biosensors)) in the title, abstract, and keywords was conducted from 2018 to January 2024. This search yielded 210 scientific papers, out of which 13 met the criteria for further in-depth analysis as depicted in Figure 1 (adapted from the current study).

### 3.1. Selection of Eligibility and Exclusion Criteria

The studies excluded from consideration fell under the following categories: reviews, book chapters, proceeding papers, conference papers, and notes; papers not related to the application of biosensors to the gut microbiota; studies that did not address biosensors for the detection of the gut microbiota and their metabolites; publications released before 2018; and those not published in English. Thirteen eligible publications were identified and further screened based on the following sub-criteria: publications related to microbial and aptamer biosensors, those focusing on the detection of microorganisms and their metabolites in the gut using electrochemical and optical biosensor approaches, and papers on methodological development and case studies. Following the application of these sub-criteria, 13 publications were selected for detailed discussion.

### 3.2. Results

The articles focused on biosensor applications for analyzing the intestinal microbiome. The search yielded 46 articles on the ScienceDirect platform, 100 articles on Scopus, and 64 articles through the PubMed search site, as indicated in the flowchart. Upon applying the eligibility criteria described in this study, 13 publications were chosen for additional analysis. The assessment centered on the methodological approaches employed to detect gut microbiota using aptamer and microbial biosensors. Data analysis showed that these 13 publications discussed two types of biosensors—8 discussed microbial biosensors while 5 focused on aptamer biosensors (Tables S1 and S2).

## 4. From the Past to Present: Biosensors Applied to the Detection of Microorganisms and Metabolites

Microorganisms and their metabolites possess a wide range of distinct characteristics that make it possible to identify them using various methods, such as traditional media, gas chromatography [34], capillary electrophoresis [35], high-performance liquid chromatography, fluorescent in situ hybridization [36], and Enzyme-Linked Immuno-Sorbent Assay [37]. Additionally, 16S rRNA sequences [35], matrix-assisted laser desorption ionization-time of flight mass spectrometry [38], and molecular methods [39] were applied. Although these methods boast excellent characteristics like high sensitivity, selectivity, and stability, they are often associated with wozhigh costs, poor automation, and require extensive sample preparation and skilled technicians. Additionally, they are time-consuming and inconvenient for rapidly identifying harmful microorganisms and metabolites, which in turn hinders the swift prevention of several diseases [12,15,40,41]. In recent years, Fourier transform infrared (FTIR) and Raman spectroscopy have been utilized for analyzing various biological materials, including saliva [42], sperm, blood [43], plasma [44], and cancer stem cells [45]. These spectroscopic methods have been instrumental in detecting microorganisms and their metabolites across different biological fields [46,47]. FTIR and Raman spectroscopy are simple, rapid, highly versatile, non-destructive, accurate, and cost-effective. They enable the conversion of chemical bonds and signals into images without the need for reagents or generating waste. These methods facilitate the detection and quantification of various biomolecules such as lipids, nucleic acids, and proteins, thereby enabling the identification of microorganisms and the characterization of diseases [46]. However, the FTIR spectra can be significantly affected by the presence of water vapor, leading to overlapping peaks and interference in the region of interest, which complicates the interpretation of results. Additionally, in Raman spectroscopy, fluorescence can cause interference. Many samples, especially biological and organic materials, can fluoresce under laser excitation, overwhelming the Raman signal and complicating the analysis. Raman spectra may display overlapping bands, making it challenging to resolve individual components. Advanced data analysis techniques, such as chemometrics, may be necessary to interpret the spectra, further adding to the complexity of the analysis [46,47]. Nowadays, new technologies have emerged, specifically the use of nanoparticles, microfluidic platforms, specific biorecognition elements, and artificial intelligence. Biorecognition element-based biosensors, such as antibodies, nucleic acids, and aptamers, permit the detection of pathogenic bacteria [15]. These methods are easy to handle, often portable, permit analysis in real-time, and are suitable for measuring biomarkers [46,47]. Biosensors produce a quantifiable and processable signal, and after recognition of specific target analytes, this signal is amplified, processed, and converted into a digital format [17]. Biosensors integrate biorecognition molecules that selectively capture target molecules, specifically antibodies, enzymes, molecular imprinted polymers, cells, nucleic acids, aptamers (DNA or RNA molecules), and alpha transcription factors in conjunction with optical (fluorescence or luminescence, surface-enhanced Raman scattering (SERS) [14,48,49,50,51,52,53], localized surface plasmon resonance (LSPR)/SPR) [54] or electrochemical transducers [7] (semi-conducting materials/nanomaterials) to convert biological signals into electrical, magnetic, mechanical, thermal or optical signals [15]. The interaction between biorecognition molecules and analytes can be categorized as bio-affinity receptors with substrate specificity, biocatalytic receptors, and hybrid receptors based on complementary sequences of DNA or RNA [55]. The transducers selected depend on the type of signals emitted by the biorecognition molecule. These have an important function to transform the biochemical signal into a quantifiable electronic signal, proportional to the analyte concentration [7,14,15,56]. Biosensors can be used for in vitro diagnosis [57], integrated biosensor devices, CRISPR-based biosensors [58], continuous monitoring, and wearable biosensors [59]. Biosensors can be applied in wide areas, specifically drug discovery [60], disease detection [59], prosthetic devices [61], water quality management [62], environmental and soil monitoring [63], toxins [64], and food quality [65]. When a biosensor is developed for commercialization, it is necessary to pay attention to the following characteristics: selectivity, sensitivity, stability, reproducibility, linearity, lower production costs, and reusability [59]. Selectivity refers to the biosensor’s ability to generate a positive result only in the presence of a specific target analyte [53,66]. This can be enhanced through specific reactions between antibodies or enzymes and the target. Sensitivity is important as biosensors should be able to detect lower analyte concentrations. The upper limit of detection and lower limit of detection (LOD) need to be established to define the range of detectable analyte concentrations [12]. Reproducibility is essential for demonstrating the precision and accuracy of the biosensor’s transducer and electronics. It ensures that the biosensor generates consistent responses in duplicate experiments, thus enhancing reliability and robustness [15]. Additionally, reusability is valuable in making biosensors sustainable and cost-effective [7,53]. Stability is also crucial, as environmental factors, temperature, receptor affinity, and bioreceptor degradation can impact the biosensor’s performance over time. Furthermore, biosensors should be capable of detecting trace concentrations of analytes, typically in the range of ng/mL or even fg/mL [23,67,68]. The limit of detection (LOD) or sensitivity is the minimum amount that can be detected by a biosensor. Finally, linearity is important for defining the resolution and range of analyte concentrations that the biosensor can effectively detect and measure. A good resolution is necessary for applications requiring the measurement of analyte concentrations over a wide range, and linearity ensures that the biosensor’s response changes linearly within the range of analyte concentrations. Recently, aptamer-based biosensors have emerged as a promising alternative to antibodies due to their higher sensitivity, stability, and selectivity. These characteristics enable them to effectively detect microorganisms [12,69,70].

## 5. Biorecognition Elements Applied to Gut Microbiome 

### 5.1. Microbial Sensor

The microbial sensor can be applied to food safety, environmental monitoring, healthcare, and industrial areas. It has a better capacity to detect and quantify biomolecules than other biorecognition sensors. Microbial biosensors can detect gut biomolecules, specifically benzoate, lactate, anhydrotetracycline, and bile acids, with higher specificity, originating a robust response [71]. Additionally, the microbial biosensor is a non-invasive, precise, and complementary tool to detect short-chain fatty acids, predicting gut dysbiosis [4,8]. The major limitations are related to the necessity of know-how in microbiology, genetics, and sensor technology; to finish the limited stability of cells over time, specifically environmental conditions such as sterilization and biocompatibility, is another limitation. Furthermore, these sensors have several advantages, such as high specificity, rapid response time, inexpensive, portability, and continuous real-time signaling [7]. An additional advantage is the application of cells over enzymes in the sensor, including overall better stability in terms of inhibitors, pH, and temperature conditions [72]. In the final analysis, it is an excellent alternative compared with enzyme receptors, which present higher costs and are time-consuming. There are several methods of immobilization applied to the microbial sensor, explicitly, cross-linking, adsorption, covalent binding, encapsulation, and entrapment. In Table S1, it is evident that the focus has been on detecting gut microbiota metabolites using fluorescence as it offers superior sensitivity and specificity. Fluorescence biosensors can detect extremely low analyte concentrations thanks to their high sensitivity to fluorescent signals. Even minor fluctuations in the concentration of the target analyte can result in significant changes in fluorescence intensity, enabling the detection of minute amounts [4,24]. This specificity is related to the use of selective fluorescent probes or fluorophores that bind specifically to the target analyte [7]. Recently, Lebovich and Andrews developed propionate and GABA sensors optimized for the probiotic bacterium *E. coli* Nissle 1917 with DNA construction or permutation of genetic parts. The EcN propionate sensor has a large 59-fold dynamic range and >500-fold increased sensitivity, while the EcN GABA biosensor has an observed 138-fold activation in EcN at biologically relevant concentrations [4]. In another representative example, five bacterial biosensors were assessed in fecal samples using the *E. coli* strains DH5αZ1 and NEB10β; three were based on cytosolic transcription factor systems, including the BenR activator and the pBEN promoter, the TetR repressor and the pTET promoter, and the LldR regulator and the pALPAGA promoter. The TcpP/TcpH and VtrA/VrtC correspond to bioreceptors for bile salts, activated via ligand-induced dimerization. Endogenous bile salts were detected by the TcpP/TcpH bile salts biosensor in fecal samples [71]. An AuNPs biorecognition element and multiple cross-displacement amplification combined with a lateral flow biosensor permitted to the detection of *Shigella* with a detection range of 10 ng–10 fg and a limit of detection of 10 fg for culture and 5.86 CFU/mL in human feces [73]. Researchers developed a microbial biosensor based on biocompatible material for the detection of acetate, applying *E. coli* immobilized on the surface of the transducer [7]. A molecular promoter and transcription factor were successfully developed in *E. coli*, utilizing the Superfolder green fluorescent protein (sfGFP) plasmid as output. This approach demonstrated exceptional sensitivity, detecting concentrations ranging from 0 to 45 mM, with a high degree of specificity for fucose in biological samples. The results suggest that this tool could be particularly useful for clinical research, especially when analyzing fecal samples or bioreactors [74]. *E. coli* DH5α was used for the propionate biosensor; afterward, the propionate sensor plasmid lacI was cloned into the pPro24 structure, including the prpR sequence, and *E. coli* BL21 was used for the butyrate biosensor, where the Ptac promoter of Ptac_sfGFP_ColE1_C or Ptac_sfGFP_ColE1_K was replaced by the PpchA promoter. Concentrations ranging from 0 to 130 mM, including those below 10 mM, were detected by the propionate biosensor. Meanwhile, the butyrate biosensor detected concentrations between 0 and 115 mM [8]. In a recent study, researchers evaluated a biosensor designed to detect human secondary bile acids and found that it was able to detect an endogenous bile acid-like molecule called Δ4-dafachronic. This finding further supports the potential use of the biosensor as a tool for identifying and monitoring bile acid-related disorders [75]. In a recent study, two biosensors were created to detect lactic acid bacteria with high levels of GABA production. One biosensor was developed in *Corynebacterium glutamicum* using the transcriptional regulator GabR, while the other was constructed in *E. coli* with auxotrophic complementation through the expression of 4-aminobutyrate transaminase (GABA-T). The biosensor based on *E. coli* exhibited a notable change in fluorescence when GABA or alanine was introduced, as compared to the negative control, in both liquid culture and intestine-like conditions [72]. In summary, microbial genetic engineering presents a high-tech prospect for detecting microorganisms and their metabolites, with the aim of preventing gut diseases. Furthermore, microbial sensors demonstrate high specificity in detecting microorganisms or microbial metabolites, as well as high sensitivity in identifying low levels of the same microbial species or metabolites. These biosensors are capable of monitoring short-chain fatty acids (SCFAs), hydrogen sulfide, and other metabolites produced by gut microbes [76,77].

### 5.2. Aptamers

Nucleic acids (NAs) are attractive therapeutics with their inherent high specificity for recognition, which promises specific interference at the root of the disease [50]. NAs are small biomolecules including plasmid DNA, siRNA, miRNA, aptamers, and oligonucleotides immensely used in the implementation of biosensors due to their easy design [78]. Aptamers are single-stranded DNA or RNA molecules (10–60 nucleotides) that are under investigation in lateral flow technology [79]. Aptamers have higher stability, specificity, affinity, and scalability of production. Moreover, they have higher chemical stability, low production cost, ease of chemical modification, low immunogenicity, and fast reproducibility in detecting a wide range of chemicals, temperature, and pH [13,69,80]. Given their tolerance for severe physical, chemical, and biological conditions, they have become increasingly important in the development of sensor devices for biomedical applications [78]. Substantial progress has been achieved over the last thirty years in developing the Systematic Evolution of Ligands by Exponential Enrichment (SELEX) technique for creating a diverse polyclonal library [26,80]. Each aptamer sequence within the library may possess distinct binding affinities and specificities toward its intended target. Polyclonal libraries are especially valuable when the target molecules display heterogeneity or when a single aptamer sequence fails to capture all variations of the target [27,80,81]. These libraries provide greater opportunities for discovering aptamers with exceptional qualities, including elevated affinity, specificity, and stability [82]. There is limited research available on the use of aptamer libraries for detecting human gut microbiota.

The studies outlined in Table S2 demonstrate the creation of aptamer libraries with the goal of developing biosensors for the detection of the gut microbiome. Xing et al., 2022 developed a high-affinity aptamer library after 14 SELEX (systematic evolution of ligands by exponential enrichment) rounds. This method efficiently discriminates *B. products*, proximally in 30 min and requires 10^8^ cells of fecal bacteria. The results showed a new possibility for rapid detection of the GM with aptamer-based biosensing applications [80]. Therefore, the detection of *R. intestinalis* isolated after seven evolution rounds through a FluCell-SELEX polyclonal aptamer library specific was shown by Xing et al., 2022 with higher fluorescence intensity (R^2^ = 0.99) and a detection limit of *R. intestinalis* for the aptamer library near 10 CFU. They also obtained a high-affinity binding entity with specificity toward *R. intestinalis* in human stool [26]. Additionally, the development of a specific polyclonal aptamer library by the fluorescence-based FluCell-SELEX for the detection of *Parabacteroides distasonis* was reported recently and obtained a coefficient of determination of 0.971 and a calculated low dissociation constant (Kd: 4.3 nM). Moreover, the polyclonal aptamer library demonstrated higher affinity, providing an accurate and sensitive recognition capacity of the aptamer to detect *P. distasonis* [81]. An enriched library of aptamers was developed using the whole-cell SELEX procedure against *Rikenella microfusus.* This allowed for labeling in fluorimetric binding assays and fluorescence microscopy, demonstrating high specificity and strong affinity (Kd = 9.597 nM after 13 rounds of selection) with a dissociation constant below 10 nm from the aptamer library [41]. In addition, an aptamer library for the easy and cost-effective detection of *A. muciniphila* was successfully developed, demonstrating an opportunity to create an innovative biosensor for GM [27]. Wang et al. described the detection of *Helicobacter pylori* with a biorecognition element, an aptamer-conjugated Fe_3_O_4_ SPMNPs. This aptasensor for the biosensing platform coupling microfluidics exhibited a low detection limit of 10, and the total time taken was 65 min [23]. In a recent study conducted by Fei et al. (2022), researchers utilized oligonucleotide probes linked with gold nanoparticles (AuNPs) in combination with *H. pylori*-specific aptamers for the analysis of stool samples to detect *H. pylori.* The assay demonstrated high sensitivity, capable of detecting even small amounts of *H. pylori* (25 CFU/mL), and exhibited increased selectivity, making it well-suited for clinical analysis. The color changes resulting from the aggregation or disaggregation of AuNPs served as an indicator of the presence of the bacterium [22]. The application of aptamers has shown several advantages, such as chemical stability and the ability to establish strong bonds and specifically bind with higher affinity to their target molecules. The recognition element is an essential part of a biosensor because it is responsible for the recognition of the analyte of interest in the samples. In summary, aptamer-based sensors represent a promising emerging technology due to the high specificity and affinity of aptamers in detecting various microorganisms and their metabolites in the gut. Notably, aptamers can be easily modified with diverse functional groups to facilitate immobilization on sensor surfaces or integration with different detection platforms.

## 6. Immobilization of Biorecognition Elements—An Important Technical Process

Immobilization consists of attaching biomolecules to or within a solid support. There are several biorecognition element immobilization methods, each with advantages and drawbacks [83]. They are divided into physical immobilization (reversible), which involves electrostatic bonding or repulsion, van der Waals forces, and hydrogen bonding, and chemical immobilization (irreversible), including crosslinking, covalent bonding, and entrapment [84]. The immobilization structure is an important part of the analysis, as it improves the sensitivity, specificity, selectivity, operational stability, detection limit, response time, and reproducibility of the signal transduction mechanism [85]. Consequently, the selection of a suitable approach depends on the transducer equipment, the biorecognition element’s nature, the analyte’s physical and chemical properties, and the operating conditions for the BS [83]. During immobilization, the biorecognition element should maintain biological activity, remain stable during the reaction, and provide proper orientation of the bioreceptor (target analyte) [84,85]. The immobilization process effectively preserves the activity of biorecognition elements on the transducer’s surface.

### 6.1. Covalent Binding 

Biomolecules, such as enzymes or proteins, are immobilized onto a solid support via covalent binding, which involves the formation of chemical bonds between the biomolecules and the support material. Generally, it is a chemical immobilization technique used to develop enzymatic biosensors, where a reaction occurs between side-chain-exposed functional groups of modified supports, inducing irreversible binding and high surface coverage [86]. This process has several advantages, including high loading capacity, thermal stability of the biomolecules, and reduced leaching, which improve the efficiency, stability, and reusability of biomolecules in several biomedical applications [87]. However, it is important to refer to some disadvantages, such as high cost, loss of structural changes in biomolecules or activity, and potential toxicity of the compounds used. For this reason, there is a necessity to select an appropriate support matrix and regulate ionic strength, pH, time, and temperature. Another parameter to improve accessibility is the addition of spacers or linkers between the support and the molecules [55]. Functional groups such as -NH_2_, -COOH, -OH, C_6_H_4_OH, and -SH can be utilized in the multipoint type of covalent immobilization. The amino, carboxylic, phenolic, sulfhydryl, thiol, imidazole, indole, and hydroxyl groups are enzyme functional groups applied in this method [85,86]. The synthesized DNA probe attaches at the 3′ or 5′ end to covalently bind to either the metal surface or the specific functional group deposited on the electrode surface during analyte analysis.

Covalent bonding in biosensors offers high robustness, specificity, and sensitivity due to the precise orientation and stable attachment of recognition elements. It provides a strong and stable attachment of biomolecules, increasing the stability of the biosensor. Additionally, covalent binding allows for multiple uses of the biosensor without significant loss of activity. To optimize this, it is important to enhance immobilization efficiency, ensure consistent and reproducible surface modification, and address potential interferences from gut microbiome samples.

### 6.2. Adsorption

Adsorption is an approach to immobilize biomolecules through hydrogen bonding, van der Waals forces, and electrostatic or hydrophobic interactions, and it is applied to the immobilization of microorganism cells, enzymes on solid surfaces, and DNA probes [88]. This method has numerous advantages, such as being user-friendly, low-cost, and retaining enzyme structure and activity. However, limited stability, difficulties in controlling orientation, enzyme leakage, and loading of the enzymes are disadvantages of this method [55]. It is possible to override these disadvantages through control of pH, ionic strength of the solution, temperature, and time of adsorption [88]. The nature and properties of the support material, and the presence of additives or modifiers, are important characteristics. The use of adsorption immobilization is a simple and cost-effective method. Compared to covalent binding, it offers lower costs. Its reversible attachment allows for easy regeneration or replacement of the sensor surface, making it applicable to a wide range of bioreceptors. However, its stability is lower than the covalent binding method due to non-covalent interactions, which may result in weaker attachment. Variability in the adsorption process can affect reproducibility and reliability, and non-specific binding can lead to potential non-specific adsorption of other biomolecules, causing background noise and reducing specificity.

### 6.3. Self-Assembled Monolayer 

A self-assembled monolayer (SAM) is an organic interphase (nanostructured) 1 to 3 nm thick, which provides precise thickness, acts as a physical barrier, and alters electronic conductivity and local optical properties [89]. When the substrate is immersed in a monolayer molecule’s solution, it is possible to observe the aggregation outstanding to the chemisorption of organic molecules on a determined substrate [90]. Therefore, it is necessary to control temperature and reaction with the substrate for some time to accomplish the assembly. This aggregation has the purpose of assembling individual molecules into highly ordered structures to obtain a desired function [91]. The molecules have different terminal groups, such as thiols, sulfides, amines, and others. Moreover, the packing density and conformation of monolayer molecules depend on the binding site of the substrate-adsorbate chemical reaction, originating intermolecular interactions (Van der Waals forces and hydrophobic interactions); consequently, it forms a monomolecular film with high orientation and density. SAM is a robust method, low-cost, and offers device flexibility, stability enhancement, and work-function modification according to the looked-for application. Moreover, this method permits immobilizing certain biomolecules in the proximity of the electrode [91]. Surface immobilization based on self-assembled monolayers (SAMs) is a powerful technique for biosensor development. It provides stable and robust attachment of bioreceptors, enhances sensor sensitivity and specificity, enables precise control over the orientation and density of immobilized bioreceptors, and can be tailored to various substrates and bioreceptors. This method is applicable in detecting pathogens and metabolites in clinical samples, such as immunoassays for cancer markers and aptamer-based sensors for small molecules. However, it is essential to ensure the formation of high-quality, defect-free monolayers. Consistent and reproducible SAM formation, as well as proper integration of SAM chemistry with the sensor substrate and bioreceptor, are crucial to achieve stable and functional immobilization.

### 6.4. Cross-Linking

The cross-linking method consists of the firm immobilization of receptors by covalent bonds. The solid substrate’s biomaterials for immobilization can be polymers or glass beads. The cross-linking method improves stability and reusability and reduces leaching of bioreceptors [83]. Moreover, this method has several disadvantages, specifically difficulty in controlling the degree, poor reproducibility, loss of activity, mechanical stability, and specificity of cross-linking [92]. Temperature, concentration of the crosslinking agent, pH, biomaterial, and choice of crosslinking agent require careful optimization for successful immobilization. Glutaraldehyde is an example of a bifunctional substance used to bind biological elements. When glutaraldehyde presents poor results, it is possible to use, for example, dextran polyaldehyde [93]. The cross-linking immobilization method offers exceptional stability by building covalent bonds through cross-linking agents. This technique is versatile and suitable for a wide range of bioreceptors, allowing for high-density immobilization. This improves the sensitivity and capacity of the biosensor. Additionally, cross-linked bioreceptors demonstrate reduced likelihood of leaching from the sensor surface, thus enhancing long-term reliability.

### 6.5. Entrapment

Entrapment is an immobilization method that involves the physical confinement of an enzyme or cell inside a porous matrix or membrane without disturbing its activity. The bioreceptor elements are combined with a monomer solution formerly polymerized to produce a gel [88]. This method can reduce enzyme denaturation and leaching, as well as improve mechanical stability. Generally, entrapment is avoided due to mass transfer limitations, lack of chemical interaction between the enzyme and polymer, and high material and equipment costs. It is possible to compensate for these problems by increasing enzyme load, changing the enzyme structure, and optimizing matrix properties [94]. The method has several advantages, such as the creation of an optimal microenvironment for the enzyme, increased stability, no need for covalent linkage, and ease of separation and reuse [55]. The support material is crucial for precise immobilization; materials like oxides, zeolites, silicates, activated carbon, polymers, and composites are widely used. Moreover, nanostructured supports like nanofibers and pristine are being developed for wide-ranging applications in the fields of fine chemistry, biofuels, and biomedicine. In summary, entrapment is a crucial immobilization method that significantly impacts biosensor performance. Biosensors have proven fundamental in several fields for their ability to employ specific biological elements, demonstrate high sensitivity in detecting low analyte concentrations, and maintain stability. By integrating the entrapment immobilization technique with biosensor technology, there is potential to enhance the detection of gut microbiomes.

## 7. Transducers

Biosensors have been developed for biological and biochemical applications. Different categories of transducer methods have been developed and are classified as electrochemical, optical, calorimetric, acoustic, and electronic biosensors [7,83]. Electrochemical biosensors are frequently classified into four groups based on the type of measurements: amperometric, potentiometric, conductometric, and impedimetric [83]. Outstanding the excellent characteristics, explicitly, selectivity, sensitivity, and bioanalysis detection capability, this method has the potential to help bio clinical groups detect microorganisms and their metabolites in the gut, therby preventing aging-related diseases [17].

### 7.1. Electrochemical Transducing

Electrochemical transducers have promising characteristics regarding autonomy, applicability, and integration of the output signal when applied to quantitative measurements and presence/pattern screening [95]. In sum, the signal in electrochemical biosensors is obtained when a biochemical reaction occurs between the bioreceptor and the target analyte, which is then measured using suitable transducers, namely amperometric, potentiometric, conductometric, and impedimetric [95], which detect the concentration of the desired analyte. Normally, electrochemical biosensors are characterized by chemically modified electrodes. Regarding aptasensors, the detection of the analyte occurs due to the increase or decrease in the electron transfer rate, called conductivity change, due to the presence of the analyte. Nowadays, there are electrochemical aptasensors that have advantages in comparison with other methods due to the use of aptamers as receptors. They have several advantages, specifically higher design flexibility, thermal stability, chemical stability, and convenience for the development of labeled electrochemical methods [83]. Furthermore, following the principle of base complementary pairing, the electrode is integrated via chemical or physical routes and is suitable for the detection of some special samples [96]. In general, electroactive substances such as enzymes and methylene blue are incorporated as tags in traditional electrochemical aptasensors because the binding of aptamers to pathogenic microorganisms cannot produce electrochemical signals by themselves [12].

#### 7.1.1. Electrochemical Point-of-Care Devices Challenges

The development of point-of-care devices has revolutionized medical diagnostics by providing rapid, accurate, and on-site testing, enabling quick decision-making and interventions. Portable electrochemical biosensors have garnered significant attention due to their ability to detect glucose, cholesterol, and infectious diseases on-site. These biosensors are known for their portability, ease of use, and real-time analysis capabilities, making them suitable for on-site and in-field applications by non-specialized personnel [97]. Recent advancements in portable electrochemical sensors have focused on improving data acquisition, processing, and communication for enhanced portability and user-friendliness, as well as continuous monitoring of physiological parameters using wearable devices [98,99]. Additionally, there has been a focus on the application of nanomaterials to enhance sensitivity and specificity, and the utilization of 3D printing technologies for creating customized and cost-effective sensor platforms. A nanotip array-based electrochemical sensing platform was developed for the detection of indole derivatives such as indole, tryptamine, and indoxyl sulfate. This approach offers rapid analysis, minimal sample preparation, and relatively low cost for analyzing microbiota-associated metabolites. The sensor exhibited excellent reproducibility (<5% RSD) and selectivity for indole derivatives, with detection limits as low as nM concentrations [99]. By integrating nanotip sensors with microfluidics and electronics, the development of portable devices for decentralized testing is feasible, holding great promise for biomedical and clinical applications. However, several challenges still need to be addressed, including improving sensor performance to detect low concentrations of analytes in complex matrices for increased sensitivity and selectivity, ensuring long-term stability and robustness of portable sensors under various environmental conditions for increased stability and durability, developing efficient data handling and analysis tools for large-scale field data, and addressing regulatory requirements for commercialization and widespread adoption [100].

#### 7.1.2. Amperometric Sensors

Amperometric transducers can be divided into three generations based on electron transport. In general, this methodology consists of passing an electric current through an electrode and detecting the analyte signal when chemical reactions occur between the analyte and the bioreceptor (enzymes, aptamers, microorganisms) [101]. Amperometric biosensors can be monoenzymatic or multienzymatic depending on the number of enzymes used. They contain pairs of redox enzymes and other enzymes that convert an analyte into a particular form, which can be oxidized in the next reaction with a redox enzyme [96]. The multienzyme is more advantageous because it consists of a cascade of multienzyme reactions capable of monitoring some clinically important metabolites, namely cholesterol, creatinine, and triglycerides. This method offers high selectivity, sensitivity, speed, reliability of measurement, compactness, and affordability for detecting biochemical metabolites (polyphenol, sulfites, glucan, L-lactate) in several areas, namely agro-food, pharmacology, and clinical diagnosis [55]. The properties of the transducers depend on the enzyme immobilization methods and the physicochemical properties of the materials used. Carbon nanomaterials and gold, used in the development of aptasensors, are crucial due to their excellent mechanical, electronic, and optical properties [23]. Aptamers were attached to the surface of AuNPs to measure the electrochemical response by amperometry in the detection of *E. coli* in fruit juice. When the target analyte binds to the *E. coli-specific* aptamer, it reduces the activity of the nanozyme, weakening the current and setting the *E. coli* detection limit to 10 CFU/mL [69]. A microbial biosensor capable of detecting acetate on the surface of Au IJP electrodes was developed. The concentration of microorganisms is immobilized on the transducer surface at approximately 10^9^ CFU mL^−1^ by electrodeposition of alginate hydrogels, and acetate is detected under aerobic conditions between 11 and 50 mM with a sensitivity of 0.035 μA h^−1^ mM^−1^, without compromising >90% bacterial or >80% mammalian cell viability [7].

In summary, amperometric biosensors offer a promising technology for monitoring the gut microbiome due to their high sensitivity, which is crucial for early diagnosis and monitoring. Moreover, real-time monitoring provides continuous data, enabling dynamic tracking of the gut environment. Furthermore, advancements in microfabrication and nanotechnology have led to the development of ingestible devices that can pass from the mouth to the gut.

#### 7.1.3. Conductometric Sensors

Conductometric biosensors allow the measurement of conductivity due to the occurrence of a biochemical/enzymatic reaction, affecting the selectivity of the method. The technique consists of determining the conductivity (reciprocal of resistivity) of a sample between two parallel electrodes [40]. These operate on low-amplitude alternating voltage, are insensitive to light, can be miniaturized, mass-produced using inexpensive technologies, and easily integrated using standard thin-film technology. Furthermore, they do not require the use of a reference electrode. Outstanding, to several reactions and mechanisms, a wide spectrum of different analytes can be determined [102]. Additionally, the driving voltage can be satisfactorily low, significantly decreasing energy consumption. It has been shown that changes in conductivity can be recorded as bacterial populations multiply non-invasively. Researchers have developed a conductometric method to monitor the growth of *E. coli* and *Staphylococcus aureus* based on magnetic analyte separation via aptamer. Compared to other bioreceptors, aptamers show better stability over a wide range of pH, ionic environments, and temperatures. In addition, they are easy to synthesize, highly resistant to denaturation, have a long shelf life, and are subject to well-controlled modifications. The following results were obtained for *E.coli* and *S. aureus* cells: in pure water, they presented linear ranges of 2.5 × 10^3^–2.5 × 10^8^ CFU·mL^−1^ and 4.1 × 10^3^–4.1 × 10^8^ CFU·mL^−1^, respectively, and in spiked tap water samples, the LODs were 2.3 × 10^4^ CFU·mL^−1^ and 4.0 × 10^3^ CFU·mL^−1^ with recoveries of 87.0–108.7% and 92.5–105.0%, respectively. In short, this process requires neither bulky, sophisticated equipment nor expensive reagents. In the future, simple analytical platforms for generalized quantitative bacteriology could be used and created [40]. To summarize, conductometric sensors are simple and robust, allowing for miniaturization and the development of small, portable devices suitable for field settings or point-of-care diagnostics.

#### 7.1.4. Potentiometric Sensors

Potentiometry is a static method (current = zero, passive/equilibrium technique) in which no current flows between the electrodes, and it is based on detecting the change in the electrical potential of an electrode when it comes into contact with the analyte [17,83]. The measurement takes place between two electrodes: indicator and reference. Potentiometric sensors have glass-coated electrodes that allow pH measurement as well as ion-selective electrodes used to measure species such as Ca^2+^, Cl^−^, K^+^, NH^4+^, H^+^, and other molecules, such as glucose and penicillin. The silver/silver chloride reference electrode system is preferred because of its stability, compatibility, environmental, and reproducibility [55]. The signal is based on the Nernst equation and is generated due to the selective partitioning of ionic species between the ion-selective membrane and the solution. This is an appropriate technique that can be applied to several areas such as medicine, environmental monitoring, agriculture, industry, and pharmaceutical sciences, as it performs the detection and quantification of several ions in wide concentration ranges [83]. Moreover, it is inexpensive, and user-friendly, and presents low time consumption, high selectivity and sensitivity, long lifetime, low detection limit, and wide concentration measurement range.

#### 7.1.5. Piezoelectric Sensors

There are two types of piezoelectricity-based sensors, namely surface acoustic wave (SAW) and quartz crystal microbalance (QCM) devices. It is an analytical method that allows recording molecular interactions on an appropriate surface—Crystal QCM—called a thin quartz disk with electrodes [83]. Outstanding, mass changes on the crystal surface, sensors rely on measuring changes in the resonant frequency of piezoelectric crystals. The QCM method is a useful analytical tool that offers a fast method to detect biomolecules. This consists of an intercalated voltage connected to the crystal surface by two electrodes, causing mechanical oscillations of the crystal [17]. The change in the oscillation frequency causes the growth of a layer on the surface of the crystal, which is proportional to the mass deposited on the crystal [83,96]. However, the high susceptibility of antibodies to chemical, thermal, and enzymatic degradations is a disadvantage in the practical application of these biosensors [59]. Researchers developed a quartz crystal microbalance (QCM) sensor for the determination of *Bifidobacterium bifidum* coupled with amplifying sandwich assays (Au nanoparticle). They observed a linear detection range between 10^3^ and 10^5^ CFU/mL with a limit of detection of 2.1 × 10^2^ CFU/mL [103]. Acoustic wave biosensors are generated by oscillating a crystal quartz plate with resonance frequencies as high as 600 MHz. This technique uses crystals without a center of symmetry that have piezoelectric characteristics. The process consists of applying an alternating electric field through a crystalline substrate that expands and contracts, originating acoustic waves that propagate along the surface of the crystal [83]. The measurement is performed when the analyte interacts with the detection surface, causing a change in the resonant frequency mainly caused by mass adsorption or viscosity changes. These are known as Rayleigh waves due to the coupling between longitudinal and shear waves [17]. The SAW sensor has several advantages, such as high ruggedness, low time consumption, high frequency among acoustic devices, and small size. Additionally, it has a quick response, great integration with different receptor materials, and can be configured in microarrays. In short, it is a sensitive and effective technology for the unmarked detection of molecular interactions. Piezoelectric sensors can be used to monitor the gut microbiome by detecting changes in mass caused by the attachment of microbial cells or biofilm formation on the sensor surface. This provides valuable information about the growth rates or activity of specific microbial species within the gut. Additionally, these sensors can be functionalized with specific recognition elements, such as antibodies or aptamers, to detect microbial metabolites like short-chain fatty acids (SCFAs) with high accuracy.

### 7.2. Optical Transducing 

Optical biosensors are powerful and versatile tools preferred in chemical and biological applications and are widely used in various fields such as biomedical, healthcare, pharmaceutical industries, environmental monitoring [62], and food safety [52]. Furthermore, they have been implemented in field applications for the monitoring of several parameters. Fluorescence, Raman scattering [46], surface plasmon resonance (SPR) [104,105], Localized (L), colorimetric, and fiber optic biosensors have been applied as optical biosensors. On the other hand, fluorescence-based detection and label-free detection are two optical biosensor procedures [53]. The optical transducer system generates an optical signal obtained by reflectance, absorption, luminescence, or transmission. Compared with other methods, optical biosensors have higher selectivity and reliability, low limits of detection (LOD), cost-effectiveness, and low time consumption. Moreover, they need a small sample and are able to monitor a target biomolecule in real time [14]. New nanoparticle materials have been developed for optical biosensors to avoid the need for sophisticated instruments and allow quick and convenient bacterial identification.

#### 7.2.1. Surface Plasmon Resonance 

Surface plasmon resonance (SPR) has been widely applied to the study of biomolecule interactions, such as DNA-DNA, DNA-RNA, peptide-protein, and protein-protein [54]. This method has several advantages, including high sensitivity to the refractive index immediately adjacent to a thin metal film. The localized surface plasmon resonance (LSPR) biosensor uses gold and silver nanomaterials due to their good trapping within conductive nanoparticles, high surface-to-volume ratio, and exceptional dielectric properties [106]. LSPR involves the interaction of the electromagnetic wave with electrons in nanoparticles of noble metals, such as Au and Ag [107]. This interaction generates localized and coherent plasmonic oscillations. As a result of this excitation, a significant increase in the near field can occur due to the high localization of the electromagnetic field beyond the diffraction limit. The shape, size, dielectric environment, composition, and distance of nanoparticles affect the intensity and frequency of the plasmonic band [54]. Furthermore, noble metals can be applied with SPR sensors to increase the sensitivity of the electromagnetic field to the changing dielectric surrounding environment. Electromagnetic fields are created due to the movement of electrons inside and outside the structure. Currently, nanostructured SPRs such as metallic nanoholes, nanorings, and nanomushroom matrices have been developed and applied as methods of biomedical detection, food and environmental monitoring. This method has the advantage of not requiring additional coupling structures, making it advantageous for multiplex detection with high throughput, miniaturization, and device portability [107,108]. In a study, researchers constructed Ω-shaped fiber optic localized surface resonance coupled with poly adenine-tailed aptamer and SH-modified gold nanoparticle tags, achieving an LOD of 108.0 CFU/mL (10 min) for the detection of high concentrations of bacteria and 7.4 CFU/mL (100 min) for bacterial trace detection, showing excellent potential in practical bacterial detection [106]. In short, the SPR method can be widely applied as biosensors and imaging agents in different areas due to its ability to use molecularly imprinted polymers, polyvinyl alcohol, silver nanoparticles, and gold-based nanoparticles.

#### 7.2.2. Surface-Enhanced Raman Spectroscopy 

Surface-Enhanced Raman Spectroscopy (SERS) is utilized for the rapid, highly sensitive, and non-destructive detection of biological and chemical analytes. This method finds application in various fields including biology, materials science, analytical chemistry, and chemical trace analysis. The major part of analytes can be detected as label-free; however, bacterial detection can be label-based (indirect) [104] or label-free (direct) [109,110]. Therefore, this method consists of a molecular vibration spectroscopy technique with enormous advantages, specifically allowing the relaxation of excitation energy at a faster rate, inducing a more stable and resilient signal, and providing a narrower peak width of about 1–2 nm [68,111]. Additionally, the negligible Raman scattering of water offers advantages over infrared spectroscopy during biological analysis. Latest, SERS has provided information about the structural molecule characteristics, resulting from an inelastic scattering process [112]. Normally, antibodies, aptamers, antimicrobial peptides, and antibiotics are used as specific capture probes for bacteria [113,114]. It is important to note that SERS can be combined with several methodologies, including chemical separation, labeling techniques, colorimetry, biological capture, and microfluidic devices. Moreover, it can be coupled with chemical analytical methods such as infrared spectroscopy, X-ray photoelectron spectroscopy, nuclear magnetic resonance, and mass spectrometry. Therefore, combining CRISPR technologies or CRISPR-associated proteins with the SERS platform presents several advantages for the rapid detection of pathogens [115]. It has been observed that the application of Au@Ag core-shell nanorods (Au@Ag NRs) in biosensor development improves the SERS signal and specific aptamer identification [105]. SERS aptasensors have been applied for the detection of several microorganisms, namely, *V. parahaemolyticus* [116], *S. aureus* [114] and *E. coli* [104]..

A novel surface-enhanced Raman spectroscopy method utilizing aptamers was developed for the detection of *S. Enteritidis.* After 6 h of enrichment, the method identified approximately 1 CFU·(10 g)^−1^ and accurately determined *S. Enteritidis* with a limit of detection of 52 CFU mL^−1^. Additionally, the method demonstrated a shorter detection time compared to other methods commonly used in pharmaceutical and food preparation standards, which typically require more than 54 to 96 h [117]. In another study, an isolation method based on aptamer-coated magnetic beads (Apt-MBs) and the label-free SERS method was developed for the rapid detection of *Escherichia coli*, *Salmonella Typhimurium*, and *Staphylococcus aureus*, identifying pathogens at a concentration level of ∼ 1 CFU in drug samples within 24 h [67]. A simple, rapid, sensitive, and high-throughput method was developed for *E. coli* O157:H7 using aptamer-modified gold nanoparticles @ macroporous magnetic silica photonic microsphere (Au@MMSPM), demonstrating a wide linear detection range (10–10^6^ CFU/mL), a low limit of detection (2.20 CFU/mL), and a reduced analysis time of approximately 110 min [70]. The aptamers exhibited a high packing density on SERS substrates, attributed to their size, which enhanced the SERS signal-to-noise ratio by reducing the overlap between the SERS signature of the aptamer and the spectral range of the target cells. Additionally, they displayed remarkable specificity, versatility, and target binding capacity, with a K D range of approximately 10^−3^ M to 10^−12^ M [12,104,114,116]. In summary, SERS with aptamer-based detection is a highly effective method that allows for the identification and quantification of microorganisms present in the gut with high sensitivity and specificity. SERS-aptamer sensors are a powerful tool, providing a significant enhancement in the Raman signal, thereby enabling the detection of microorganisms at very low concentrations. Moreover, they offer high specificity due to their ability to tightly and selectively bind to their targets, as well as rapid detection, which is crucial in situations requiring timely diagnosis or intervention. Lastly, the platform can be adapted to detect a wide range of microorganisms by simply changing the aptamer sequence.

#### 7.2.3. Fiber Optic Biosensors 

Fiber optic biosensors (FOBs) have been applied to pathogens and toxic substances detection in the environment, enzyme measurement, food quality detection, and cancer biomarker detection. Moreover, they rely exclusively on optical transduction mechanisms and consist of a fiber core, shell, metal cladding, and a sensitive layer for detecting of target biomolecules. The principle used by FOBs is total internal reflection, which involves the passage of light through the core of the fiber, where it is surrounded by a coating [59]. The core is usually doped with germanium, which makes the refractive index slightly higher than that of the cladding. Biosensor elements, such as aptamers, microorganisms, and enzymes are immobilized in the fiber core to react with the target biomolecule. Fiber optic biosensors have several advantages, such as being cheaper, smaller, user-friendly, environmentally robust, and highly sensitive and specific to changes in the effective refractive index at the fiber’s surface, and also offer remote sensing capabilities. Furthermore, this method can be utilized with minimally invasive sampling techniques, detect multiple analytes, and provide immediate information on the gut. Its compact and portable design makes it suitable for both on-site and portable applications, including potential use in clinical settings [118].

Fiber optic biosensors, including fluorescence-based biosensors, absorbance-based biosensors, surface plasmon resonance (SPR) biosensors, and evanescent wave biosensors, can detect gut microbiota. Fluorescence-based biosensors offer high sensitivity and specificity for surface detection, while SPR biosensors are highly sensitive to changes in the refractive index near the sensor surface and enable label-free detection, making them suitable for detecting biomolecular interactions. Evanescent wave biosensors are well-suited for detecting changes in microbial composition and activity near the fiber surface. Evanescent wave fiber optic biosensors can identify microorganisms and toxins in minutes from complex matrix samples, improving detection sensitivity, selectivity, and speed [119].

Researchers have developed a portable borosilicate glass fiber optic biosensor for detecting *E. coli* by applying changes in the refractive index of glass fiber [66]. Other investigators showed that a single-mode conical multimode single-mode fiber optic biosensor was able to detect *Salmonella Typhimurium* with good sensitivity [53]. Fiber optic biosensors present a highly sensitive and specific method for the real-time monitoring of gut microbiota, holding potential for the detection of metabolic disorders, inflammatory diseases, and infections. These biosensors can identify microorganisms and their metabolites, such as short-chain fatty acids and gases, offering valuable insights into the health status of the gut microbiome. By targeting unique genetic markers and surface antigens, they can be further refined to identify specific microorganisms within the gut microbiota. Additionally, this approach allows for the real-time tracking of dynamic changes in microbiota composition in response to dietary modifications, including probiotics and prebiotics. Thus, fiber optic biosensors have the potential to facilitate early diagnosis and personalized treatment, as well as to offer timely feedback on treatment efficacy and disease progression for chronic conditions. Fiber optic biosensors offer high sensitivity, robustness, and real-time monitoring, making them valuable for modern sensing and diagnostic technologies. Their potential for integration on a single chip facilitates new discoveries and enhances disease diagnosis and management [120]. However, challenges arise when applying these biosensors to the gut microbiota, including issues like biofouling and signal degradation due to long-term use in the gut environment. Accurate interpretation of the complex data generated by these biosensors requires data analysis and machine learning tools. Extensive clinical studies are necessary to validate the reliability and efficacy of fiber optic biosensors in the gut microbiota. Additionally, regulatory approval and compliance with medical device standards are crucial for clinical adoption.

## 8. Challenge and Future Perspectives: Why Is There a Continuous Need for Developing Biosensors for the Gut?

### 8.1. Developing Aptamer-Based Biosensors for the Detection of the Human Gut Microbiome 

Aptamer-based biosensors show potential for detecting the highly diverse human gut microbiome. However, challenges arise from interference by gut components and the need to develop efficient sample preparation methods. These methods should preserve target molecules and eliminate interfering substances, such as enzymes, proteins, and polysaccharides. Additionally, selecting aptamers with high specificity and sensitivity for similar target molecules or closely related metabolites poses a challenge, as does developing stable aptamers for detection under alkaline pH conditions and in high ionic strength environments. Efficient signal conversion and integration into portable devices, as well as consistent and reproducible immobilization of aptamers on sensor surfaces without loss of binding activity, are further hurdles to overcome. However, the improvement and development of aptamer libraries may open new opportunities for effectively monitoring microbial abundance in the human gut microbiome. It was observed that aptamer-based biosensing showed promise for the fast and reliable monitoring of *Blautia producta* in the human gut microbiome [80]. SELEX, which stands for Systematic Evolution of Ligands by Exponential Enrichment, is a time-consuming and labor-intensive process for selecting aptamers [27]. A major limitation in developing aptamer gut biosensors is the lack of high-quality aptamers for clinically important targets, such as a set of different microorganisms, and the need for extensive testing in clinical samples to establish reliability and accuracy. However, a split aptamer-based sandwich-type biosensor has been developed for on-site environmental monitoring. This biosensor shows potential for the facile and rapid detection of small molecular targets, specifically streptomycin [121]. The development of aptamer-based biosensors for gut health faces several translational challenges. These include the need for rigorous testing and validation to meet regulatory standards for clinical use. Additionally, demonstrating consistent performance across different populations and environments is crucial. Scalable and cost-effective manufacturing processes need to be established to ensure commercial viability while maintaining high quality and consistency in large-scale production. Furthermore, it is essential to incorporate user-friendly design elements for both patients and healthcare professionals and to provide clear data interpretation for effective use in clinical settings. In summary, the primary challenge lies in transitioning aptamer-based biosensors from the laboratory to the market.

### 8.2. Developing Alternative Detection Platforms for the Complex Matrix—Gut Microbiome

Electrochemical and optical sensors play a crucial role in various biomedical applications, including continuous glucose monitoring, neurotransmitter detection, and the analysis of small molecules. In general, electrochemical sensors may produce inaccurate readings when several microbial species are present and produce multiple metabolites. Consequently, the presence of multiple analytes can complicate the interpretation of the sensor’s output and cause cross-reactivity. Fecal samples can cause surface fouling on electrochemical and optical sensors due to the presence of proteins, lipids, and other macromolecules, leading to decreased sensitivity and reproducibility [83]. Additionally, electrochemical interference due to the presence of ions and molecules can result in background noise or false positives, posing a challenge to obtaining accurate and reliable data.

Conductometric sensors, employing changes in electrical conductivity due to the presence of a target analyte, offer promise in monitoring gut health. In fecal samples, conductometric sensors may exhibit cross-reactivity to a broad spectrum of organic and inorganic compounds, leading to false positives. Additionally, the sensors’ limited selectivity in measuring overall conductivity changes can make it difficult to accurately identify specific biomarkers or pathogens, particularly if their conductivity signatures are similar. This reduces the sensors’ precision and reliability in providing accurate data [40,102].

Potentiometric sensors, which measure the voltage difference between a working electrode and a reference electrode in response to a specific analyte, face certain drawbacks and challenges. These sensors are designed to react to specific ions, making it challenging to isolate the target analyte’s signal, especially in the presence of high concentrations of interfering substances. The use of ion-selective electrodes to detect specific ions can lead to limited selectivity and sensitivity in fecal samples, resulting in decreased accuracy and potential cross-sensitivity to other ions. Additionally, the sensitivity of potentiometric sensors to small changes in ion concentration can be compromised in highly variable fecal samples, where large fluctuations in ion concentrations are common [60].

On the other hand, voltammetric sensors utilize carbon-based nanomaterials to detect and quantify specific biological compounds such as glucose, dopamine, proteins, and enzymes by measuring the current. This sensitive and selective method can detect low concentrations. Protein-based voltammetric biosensors can be developed by reconfiguring the flow of information through de novo designed protein switches, allowing for modular design and sensitivity in detecting various analytes. However, biological samples can potentially interfere with the measurement and accumulate on the electrode surface, affecting sensor performance. Therefore, ensuring accurate and reproducible results requires careful calibration and validation.

Piezoelectric sensors offer unique capabilities for detecting and monitoring various analytes. However, the presence of multiple species can complicate the interpretation of sensor data, making it challenging to attribute changes in mass or frequency to specific microbes. Additionally, mass changes associated with microbial interactions or growth in the gut may fall below the sensor’s detection threshold, making it difficult to distinguish from background noise. Another issue is the lack of specificity in piezoelectric sensors, as they may measure changes that are not specific to individual microorganisms. This lack of specificity makes it challenging to identify and quantify individual microorganisms within the gut microbiome. Moreover, interference from non-microbial components, such as food particles, fibers, and other organic matter, can interfere with the sensor’s measurements, producing signals similar to those caused by microbial changes, leading to false positives or inaccurate data.

Surface Plasmon Resonance (SPR) is a powerful method known for its sensitivity and label-free detection properties. New amplification strategies utilizing gold nanostars and nanoparticles have been developed to enhance sensitivity and lower the limit of detection [54]. However, fecal samples can cause surface fouling, leading to decreased sensitivity and reproducibility. Consequently, self-assembled monolayers, plasmonic coupling, and shape complementarity on nanoparticle surfaces have been utilized to achieve high selectivity. The integration of SPR and LSPR technologies into Point-of-Care Devices has challenges related to miniaturization and robustness, consequently, microfluidic and multiplexed advanced devices are being developed. Furthermore, transport limitations complicate the detection of low-concentration analytes.

Surface-Enhanced Raman Spectroscopy (SERS) is an extremely sensitive analytical technique that enhances Raman scattering by molecules absorbed on rough metal surfaces or nanoparticles. However, the presence of fecal matter can lead to overlapping signals and spectral noise, making it challenging to accurately identify specific microorganisms. Additionally, the consistency and reproducibility of the SERS substrate are crucial for reliable results, and variations in sample preparation can lead to inconsistent outcomes. While SERS offers detailed qualitative information about molecular composition, its effectiveness is reduced in heterogeneous samples, resulting in significant variation in the enhancement factor and complicating quantitative analysis [46]. As a result, SERS is generally not suitable for large-scale studies of the gut microbiome that involve analyzing numerous samples or comprehensively profiling microbial communities. Additionally, this method can attain extremely low detection limits. However, the sensitivity may be impacted because large biomolecules need to access a nanogap “hot spot” between metal nanostructures, which may not always be feasible [122].

The exact nanoarchitecture of the surface of the fiber optic biosensor, including the density and orientation of bioreceptors, is not fully understood, which could impact the biosensor’s performance. Additionally, ensuring that fiber optic sensors are biocompatible, accurate, and reliable for in vivo measurements presents a significant challenge in maintaining their performance. It is important to note that optical biosensors require specific probes to capture and detect targets; producing and validating these probes can be time-consuming and may not be feasible [53,66]. Additionally, they are expensive and require specialized training to operate.

## 9. Conclusions

In recent years, biosensor technologies have become increasingly important in biomedical diagnostics and other fields [115,123]. Although there is a scarcity of research on the use of aptamers for detecting human gut microbiota, they exhibit potential for developing precise, resilient, and cost-efficient diagnostic techniques for monitoring the gut microbiome. This has the potential to decrease healthcare expenses and minimize potential harm to the human body. The presence of various microorganisms and their metabolites in the gut has been linked to a range of diseases, including obesity, diabetes, cancer, liver, and neurodegenerative diseases. Currently, it is known that gut microbiota could be a potential source for novel therapies.

The rapid detection of an abundance of specific gut microbiota is crucial for preventing potential diseases. Recent developments in biosensor design have led to enhanced sensitivity, specificity, and multiplexing capabilities for the detection of key analytes in complex biological matrices.

In recent years, there have been significant advancements in the development of assays based on aptamers and/or aptamer libraries for the analysis of microorganisms and metabolites [26,80,81]. These assays demonstrate high sensitivity and specificity, especially at concentrations below the specified limit.

The integration of nanomaterials, such as gold nanoparticles [104,105], graphene, and carbon nanotubes, in the development of aptasensors and bioassays has significantly improved sensitivity and specificity [124].

Additionally, the FluCell-SELEX method has significantly enhanced aptamer screening, making the process more cost-effective, convenient, and less time-consuming. Advancements in molecular biology, sequencing technologies, and material sciences have driven the progress and utilization of biosensors [27]. SERS [117] and CRISPR [115] have shown potential for detecting microorganisms and metabolites. These analytical methods provide a significant advantage in detecting microorganisms and their metabolites at concentrations below the maximum limit.

It is important to note that aptasensors are considered new clinical diagnostic tools for the rapid detection of intestinal bacterial abundance [104,105]. These sensors can be used to monitor the effectiveness of future supplementation strategies involving promising intestinal probiotics. Finally, this application can play a key role in reducing healthcare costs and minimizing potential harm to the human body.

## Supplementary Materials

The following supporting information can be downloaded at: https://www.mdpi.com/article/10.3390/bios14090423/s1; Table S1: Research on identifying metabolites generated in the gut by microbiota with microbial sensors; Table S2: FluCell-SELEX aptamers for the development of biosensors. References [4,7,8,26,27,40,71,72,74,76,77,80,81] are cited in the supplementary materials.
