# Supplementary material for: Are Aptamer-Based Biosensors the Future of the Detection of the Human Gut Microbiome?—A Systematic Review and Meta-Analysis
Source: Biosensors (Basel). 2024 Sep 2;14(9):423. doi: 10.3390/bios14090423 (PMC11430143; doi:10.3390/bios14090423)
Supplement: Supplementary file 1 [file biosensors-14-00423-s001.zip › biosensors-3150718-supplementary.pdf]

Table 1. Research on identifying metabolites generated in the gut by microbiota with microbial sensor.

Target analyte	Sample	Bioreceptor	Transducer	Main results and conclusions	Ref
Acetate	Stool	*Escherichia coli*	Amperometric	In aerobic conditions, acetate was found to be present in the range of 11 to 50 mM, with a sensitivity of 0.035 µA h^{-1} mM^{-1}. High concentrations of approximately 10^9 cfu mL^{-1} are immobilized on the transducer surface.	[1]
Propionate and butyrate	Medium	*Escherichia coli*	Fluorescense	The propionate biosensor functioned within a range of approximately 0 to 110 mM SCFA, with a limit of detection from 0 to 20 mM and a sensitivity of 0 and 130 mM for propionate. The butyrate biosensor functioned within a range between 30 to 90 mM SCFA, with a limit of detection from 0 to 30 mM and a sensitivity of 0 and 115 mM for butyrate.	[2]
γ-aminobutyric acid (GABA)	Medium	*Corynebacterium glutamicum* *Escherichia coli*	Fluorescense		[3]
Free Fucose	Medium	*Escherichia coli*	Fluorescense	*E. coli* with a high copy plasmid containing a reporter sfGFP, a fucose promoter, and FucR showed higher sensitivity and specificity, with a linear range between 0–45 mM at low (0–3 mM) and high concentrations (0–50 mM) with good resolution.	[4]
Gamma-Aminobutyric Acid and Propionate	Medium	*Escherichia coli*	Fluorescense	EcN propionate sensor has a large 59-fold dynamic range, a 5–30 mM propionate of limit detection, and >500-fold increased sensitivity. GABA sensor obtained a 138-fold activation and a limit of detection of 50 mM GABA.	[5]
Benzoate, lactate, anhydrotetracycline and bile acids	Stool	*Escherichia coli*	Fluorescence	The BenR-pBEN biosensor presented a limit of detection of 80 µM. The bacteria encapsulation in alginate beads gave at least 1.3 × 10^9 CFU/mL of live bacteria	[6]
Indole	Medium	*Pseudomonas putida* KT2440 *Escherichia coli* *Cupriavidus necator*	Fluorescense	The biosensor utilizing the gene expression system PpTrpI/PPP_RS00425 demonstrated a significant response within the concentration range of approximately 0.4 to 5 mM. It exhibited an impressive inducible fold increase of up to 639.6 by indole.	[7]
Thiosulfate and nitrate	Stool	E. coli Nissle 1917	Fluorescense	Observations showed that the biosensor is capable of detecting elevated nitrate levels during intestinal inflammation. Additionally, by implementing a Boolean AND gate, it was possible to simultaneously detect two inflammation biomarkers, nitrate and thiosulfate.	[8]

Table 2. FluCell-SELEX Aptamers for the development of biosensors

Analyte	Sample	Primer Sequence	Ref
Rikenella microfusus-Specific	Medium	5′-Cy5-TAGGGAAGAAGGAGAGAGATGATA-3′<br>5′-phosphate-TCAGTGTCATGTACTAGTCAA-3′	[9]
Blautia producta	Medium and Stool	Cy5-TAGGGAAGAGAAGGACATATGAT<br>Biotina-TCAAGTGGTCATGTACTAGTCAA	[10]
Roseburia intestinalis	Medium and stool	5′-Cy5-TAGGGAAGAGAAGGACATATGAT-3′<br>5′-Phosphate-TCAAGTGGTCATGTACTAGTCAA-3′	[11]
Parabacteroides distasonis	Medium	5′-Cy5-TAGGGAAGAGAAGGACATATGAT-3′<br>5′-biotin-TCAAGTGGTCATGTACTAGTCAA-3′	[12]
Akkermansia muciniphila	Medium and mice	5′-[Cy5]-TAGGGAAGAGAAGGACATATGAT-3′<br>5′-Biotin-TCAAGTGGTCATGTACTAGTCAA-3′	[13]

Reference

[1] Forner, E.; Ezenarro, J.J.; Pérez-Montero, M.; Vigués, N.; Asensio-Grau, A.; Andrés, A.; Mas, J.; Baeza, M.; Muñoz-Berbel, X.; Villa, R.; et al. Electrochemical Biosensor for Aerobic Acetate Detection. Talanta 2023, 265, 124882. https://doi.org/10.1016/j.talanta.2023.124882.

[2] Serebrinsky-Duek, K.; Barra, M.; Danino, T.; Garrido, D. Engineered Bacteria for Short-Chain-Fatty-Acid-Repressed Expression of Biotherapeutic Molecules. Microbiol. Spectr. 2023, 11, e0004923. https://doi.org/10.1128/spectrum.00049-23.

[3] Rehman, A.; Di Benedetto, G.; Bird, J.K.; Dabene, V.; Vadakumchery, L.; May, A.; Schyns, G.; Sybesma, W.; Mak, T.N. Development of a Workflow for the Selection, Identification and Optimization of Lactic Acid Bacteria with High γ-Aminobutyric Acid Production. Sci. Rep. 2023, 13, 13663. https://doi.org/10.1038/s41598-023-40808-z

[4] Nuñez, S.; Barra, M.; Garrido, D. Developing a Fluorescent Inducible System for Free Fucose Quantification in Escherichia coli. Biosensors 2023, 13, 388. https://doi.org/10.3390/bios13030388.

[5] Lebovich, M.; Andrews, L.B. Surveying the Genetic Design Space for Transcription Factor-Based Metabolite Biosensors: Syn-thetic Gamma-Aminobutyric Acid and Propionate Biosensors in E. coli Nissle 1917. Front. Bioeng. Biotechnol. 2022, 10, 938056. https://doi.org/10.3389/fbioe.2022.938056.

[6] Zúñiga, A.; Muñoz-Guamuro, G.; Boivineau, L.; Mayonove, P.; Conejero, I.; Pageaux, G.-P.; Altwegg, R.; Bonnet, J. A Rapid and Standardized Workflow for Functional Assessment of Bacterial Biosensors in Fecal Samples. Front. Bioeng. Biotechnol. 2022, 10, 859600. https://doi.org/10.3389/fbioe.2022.859600.

[7] Matulis, P.; Kutraite, I.; Augustiniene, E.; Valanciene, E.; Jonuskiene, I.; Malys, N. Development and Characterization of In-dole-Responsive Whole-Cell Biosensor Based on the Inducible Gene Expression System from Pseudomonas Putida KT2440. Int. J. Mol. Sci. 2022, 23, 4649. https://doi.org/10.3390/ijms23094649.

[8] Woo, S.-G.; Moon, S.-J.; Kim, S.K.; Kim, T.H.; Lim, H.S.; Yeon, G.-H.; Sung, B.H.; Lee, C.-H.; Lee, S.-G.; Hwang, J.H.; et al. A Designed Whole-Cell Biosensor for Live Diagnosis of Gut Inflammation through Nitrate Sensing. Biosens. Bioelectron. 2020, 168, 112523. https://doi.org/10.1016/j.bios.2020.112523.

[9] Zhang, X.; Wang, X.; Yang, Q.; Jiang, X.; Li, Y.; Zhao, J.; Qu, K. Conductometric Sensor for Viable Escherichia coli and Staphy-lococcus aureus Based on Magnetic Analyte Separation via Aptamer. Microchim. Acta 2020, 187, 43. https://doi.org/10.1007/s00604-019-3880-0.

[10] Xing, H.; Zhang, Y.; Krämer, M.; Kissmann, A.-K.; Henkel, M.; Weil, T.; Knippschild, U.; Rosenau, F. A Polyclonal Selex Ap-tamer Library Directly Allows Specific Labelling of the Human Gut Bacterium Blautia Producta without Isolating Individual Aptamers. Molecules 2022, 27, 5693. https://doi.org/10.3390/molecules27175693.

[11] Xing, H.; Zhang, Y.; Krämer, M.; Kissmann, A.-K.; Amann, V.; Raber, H.F.; Weil, T.; Stieger, K.R.; Knippschild, U.; Henkel, M.; et al. A Polyclonal Aptamer Library for the Specific Binding of the Gut Bacterium Roseburia Intestinalis in Mixtures with Other Gut Microbiome Bacteria and Human Stool Samples. Int. J. Mol. Sci. 2022, 23, 7744. https://doi.org/10.3390/ijms23147744.

[12] Xing, H.; Kissmann, A.-K.; Raber, H.F.; Krämer, M.; Amann, V.; Kohn, K.; Weil, T.; Rosenau, F. Polyclonal Aptamers for Specific Fluorescence Labeling and Quantification of the Health Relevant Human Gut Bacterium Parabacteroides Distasonis. Microor-ganisms 2021, 9, 2284. https://doi.org/10.3390/microorganisms9112284.

[13] Raber, H.F.; Kubiczek, D.H.; Bodenberger, N.; Kissmann, A.-K.; D'souza, D.; Xing, H.; Mayer, D.; Xu, P.; Knippschild, U.; Spellerberg, B.; et al. FluCell-SELEX Aptamers as Specific Binding Molecules for Diagnostics of the Health Relevant Gut Bac-terium Akkermansia Muciniphila. Int. J. Mol. Sci. 2021, 22, 425. https://doi.org/10.3390/ijms221910425.
